# Nigerian response to the 2014 Ebola viral disease outbreak: lessons and cautions

**DOI:** 10.11694/pamj.supp.2015.22.1.6490

**Published:** 2015-10-10

**Authors:** Obinna Ositadimma Oleribe, Mary Margaret Elizabeth Crossey, Simon David Taylor-Robinson

**Affiliations:** 1 Excellence & Friends Management Care Centre (EFMC), Abuja, Nigeria; 2 Hepatology Unit, Imperial College London, 10th Floor, QEQM Building, St Mary's Hospital Campus, South Wharf Road, W2 1NY, London, United Kingdom; 3 Department of Medicine, Jos University Teaching Hospital, 2 Murtala Mohammed Way, Jos, Plateau State, Nigeria

**Keywords:** Ebola viral disease (EVD), West Africa, Nigeria, EFMC

## Abstract

The Ebola virus disease outbreak that initially hit Guinea, Liberia and Senegal in 2014 was projected to affect Nigeria very badly when the first case was reported in July 2014. However, the outbreak was effectively and swiftly contained with only eight deaths out of 20 cases, confounding even the most optimistic predictions of the disease modelers. A combination of health worker and public education, a coordinated field epidemiology and laboratory training program (with prior experience in disease outbreak control in other diseases) and effective set-up of emergency operations centers were some of the measures that helped to confound the critics and contain what would have been an otherwise deadly outbreak in a densely populated country with a highly mobile population. This article highlights the measures taken in Nigeria and looks to the translatable lessons learnt for future disease outbreaks, whether that be from the Ebola virus or other infectious agents.

## Introduction

The case of Ebola reported from Guéckédou, Guinea, near the border with Liberia and Sierra Leone would have gone unnoticed, except that it led to the worst Ebola Viral Disease (EVD) outbreak the world has ever known, infecting more than 23,729 people and causing the avoidable deaths of over 9,604 with Case Fatality Rate (CFR) of 40.47 as at February 22nd, 2015 [1]. Although the current outbreaks affected nine countries in three continents, it was worst in the West African nations of Guinea (Cases = 3,155; Deaths = 2,091, CFR = 66.28%); Liberia (Cases = 9,238; Deaths = 4,037, CFR = 43.7%); and Sierra Leone (Cases = 11,301; Deaths = 3,461, CFR = 30.63%); cumulatively accounting for 23, 694 cases and 9589 deaths (i.e. 99.8% of all cases and deaths [1]. EVD was identified in Nigeria in July 2014. However, within a few weeks, it was curtailed through interdisciplinary collaboration, intensive case management, detailed contact tracing, and active port health services. Using isolation, quarantine and supportive management of the infected, case fatality rate was kept at 40% as eight out of the 20 infected individuals succumbed to the virus including several health workers [2]. The quick and decisive management of EVD in Nigeria came as a surprise to several world leaders as this completely destroyed all mathematical projections on the current outbreak, especially in Nigeria. What has not been fully documented was the process and strategies that led to the prompt control of an outbreak that would have potentially overshadowed the magnitude of the current Guinea, Liberia and Sierra Leone outbreak. In all, 20 cases were recorded in Nigeria with eight deaths with a CFR of 40%. This was higher than that of Sierra Leone, but lower than Liberia and Guinea [1]. The outbreak affected primarily two cities - Lagos and Port Harcourt; and more than 890 persons were followed up, isolated or quarantined within the period [2]. How did Nigeria succeed in this, when many thought local strategies would fail? What structures did Nigeria have or put in place that made for this historic success story? In this short commentary, we will be discussing the Nigerian success story and lessons learned from the outbreak.

### Ebola scare in Abuja

At the height of the 2014 EVD outbreak in Nigeria and with much publicity about failures of care in Guinea, Sierra Leone and Liberia, a possible EVD case was seen in Abuja, the Federal Capital Territory (FCT) of Nigeria on August 20th, 2014. The staffers of a private facility in Abuja, following sensitization training, suspected a woman referred to their clinic for treatment as a suspected EVD case [3]. The 38 year old female was a single mother of two who travelled from Lagos Nigeria (where the index case - Patrick Sawyer from Liberia - was diagnosed and died), and was manifesting some clinical signs and symptoms of EVD, such as sudden onset of fever, vomiting, facial swelling, redness of the eyes and face, bleeding from facial sores and several other symptoms. Although there was no direct contact with known cases of EVD, the staff isolated the patient, and called for support from Excellence & Friends Management Care Center (EFMC), a public health outfit in Abuja Nigeria. EFMC immediately contacted the Federal Ministry of Health (FMOH), Nigeria Center for Disease Control (NCDC) and the FCTA Epidemiology Unit. The promptness of isolation, involvement of key stakeholders and professional review of the case by trained public health experts was similar to what was seen in several parts of Nigeria due to mass public enlightenment, effective use of media and proper outbreak investigation leadership by the various Ministries of Health across the nation. Personal Protective Equipment (PPE) were released by government officials to the private hospital facility to safeguard the health and well-being of the staff of the center, blood samples were collected for investigation and patient was placed on supportive treatment. When the laboratory result came out a few days later and was negative for EVD, it was fortunate for all concerned, but also a great public health lesson, as everyone involved was better informed of the immediacy of EVD management, and prevention practices.

### Lessons from Nigeria experience

Nigeria, with a population of more than 174 million freely mobile people, who easily connected to the rest of the world, would have been an excellent culture medium for such a deadly disease. However, the nation prevented community transmission of the virus which led to the early control and nullification of the various mathematical models of EVD in Nigeria. Although the general health system in Nigeria is still weak, and will benefit from a lot of reforms and structural changes, Nigeria's success could be traced to five major strengths of the health systems which Nigeria, unlike Guinea, Liberia and Sierra Leone had.

The Nigerian Center for Disease Control (NCDC): health in Nigeria is the primary responsibility of the Federal Ministry of Health (FMOH) at the Federal level, State MOH and the Local Health Committee at the State and Local Government Areas respectively. The Nigerian CDC was a brain child of a Harvard-trained Nigerian public health physician and implemented by the former US CDC Country Director in partnership with the FMOH. This unit became the key coordinating unit for the EVD outbreak. Such a body is absent in all three countries where EVD had a field day. Moreover, Liberia and Sierra Leone were just recovering from civil wars, and are both victims of monetary policies that do not encourage employment of health workers, building of sustainable health systems, and innovations that support health system development.

The Nigeria Field Epidemiology and Laboratory Training Program (NFELT): the NFELTP was established in Nigeria in 2008 by US CDC and the FMOH to train field epidemiologists. Since inception, hundreds of Nigerian health workers have been trained either in the three to six months short courses or the two year long courses [4]. These trainings had 75% field components which saw the trainees immediately putting into practice what they learned in the field, thereby building competences in field epidemiology. Within their training, they were supported to execute several outbreak investigations including cholera, Lassa fever and lead poisoning with close mentoring and supervision. Through this work, they were able to develop skills on various aspects of outbreak investigations, contact tracing and report development and dissemination; skills that were very relevant to EVD investigation. Such a specialized cadre of health workers was completely lacking in the three most affected countries of Guinea, Liberia and Sierra Leone, as they were yet to set up their field epidemiology training programs. The cohorts of those trained were easy to reach public health workers along with willing Nigerian volunteers, ready to make a difference. This provided more than enough human resources for health as they worked selflessly to help control the dangerous outbreaks. Another major strength of the Nigerian NFELTP group was the maximization of the interdisciplinary approach. The NFELTP was the first cohort ever to apply the One Health concept in full as the program had three main tracks: Epidemiology, Laboratory and Veterinary work strands [4]. The combination of these three vital themes became more important during zoonotic disease outbreaks like EVD, Lassa fever and Marburg fever. As the team had worked together prior to the 2014 EVD outbreak to manage other public health emergencies, it was easy for them to work together to curtail the EVD challenge in Nigeria.

Functional state epidemiology units: the quick and prompt response of the Lagos State Epidemiology Unit was very instructive, as it helped to localize the epidemic and ensure cordoning of the virus within a specific region, thereby preventing community transmission. When the outbreak occurred in Port Harcourt, the local health ministry was pro-active. The immediate involvement of the State Government, quick release of funds, mobilization of skilled health workers and the establishment of Ebola Emergency Operations Centers (EEOC) were instrumental to the effective control of the epidemic. These units took responsibility for the control of the outbreak in each state, preventing spread, communicating findings and educating the people on best steps to take to prevent infection. In addition, regionally-based non-governmental organizations (NGOs) and private sector partners participated in public education, acquisition and supply of personal protective equipment (PPE), and provision of hand washing facilities in health-care and work sites with distribution of hand sanitizers for health workers.

Use of Emergency Operations Center (EOC): the decision of the Nigerian Federal Ministry of Health and all stakeholders to use an EOC was instrumental to the rapid respond to the outbreak [5]. The EOC was populated with professionals who had relevant skills and expertise in the management of similar outbreaks, such as Lassa fever. The EOC made investigation of the index patient and all ensured that exposed contacts were better coordinated, as multiple response teams in several cities in Nigeria had easy flow of communication, enhanced situation awareness and were able to assess needed logistics with minimal challenges [5]. The use of the EOC also allowed the full participation of global helpers, including from the World Health Organization (WHO), Doctors without Borders, and the US Centers for Disease Control and Prevention (CDC), who also played key roles in control exercise. It also facilitated unified control and coordination of the entire disease outbreak process, seamless transfer of information and daily meetings and updates which informed definite response at every stage of the outbreak.

Support from local, national and international partners: EVD remains a global emergency in 2015. Although this was not the case initially in Guinea where it took several months before the entire world was awakened to the challenges of the outbreak, Nigeria's experience was different. Before the first case was registered in Nigeria on July 20, 2014, through an acutely ill traveler from Liberia who arrived at the international airport in Lagos, and was confirmed to have EVD after being admitted to a private hospital in Lagos, the entire world was already aware of the epidemic and equipped to handle it [5]. Although the index patient exposed about 72 people at the airport and the hospital to the virus, within hours of the confirmation of the disease, the Nigerian Federal Ministry of Health declared an Ebola emergency. This singular step helped mobilize all relevant resources to stop the epidemic earlier than anyone could imagine. It also allowed for prompt involvement of all relevant global bodies who contributed to the Nigerian success story, especially as the disease was already recognized by WHO as a public health emergency [6].

### Cautionary tales from the Ebola viral disease in Nigeria

While the entire world - including Nigerian health officials were focusing on EVD, women and children were dying from malaria, measles, and other easily preventable communicable diseases. In some Nigerian states, funds earmarked for other health initiatives were diverted to EVD control processes to establish EVD EOC, train health workers as EVD control officers, as well as to acquire relevant personal protective equipment. In addition, fear of EVD in Nigeria led to the classical “salt water and salt bath” therapy which resulted in the hospitalization and or death of several people [7, 8]. However, this also highlighted the role of social media in public health and health education, which when properly harnessed could lead to enhanced health literacy, better health seeking behavior and improved public health outcomes. The national response enhanced port health, individual personal hygiene with proper hand washing practices and improved infection control practices in most hospitals which reduced nosocomial infections. Post-EVD outbreak, these practices were lost and Nigerians returned to usual practice prior to the outbreak. If public awareness was sustained, if hand hygiene could be linked to other diseases other than EVD alone, maybe the gains of the outbreak control program will not be completely lost.

## Conclusion

It is commendable that Nigeria was able to manage the imminent challenge very well. According to a local health magazine, the outbreak revealed that the **Nigerian Center for Disease Control (NCDC)**: the use of **personal protective equipment (PPE)** is a rarity in Nigerian hospitals, whether publically or privately funded; the **Nigeria Field Epidemiology and Laboratory Training Program (NFELTP)**: Nigerians prefer portable hand sanitizers because very few public hospitals have running water; **functional state epidemiology units:** and Nigerian authorities had not paid sufficient attention to infectious diseases outbreaks hitherto [9]. However, beyond these issues, lessons learned from EVD offer the portent for continuation of improved infection control practices, an interdisciplinary approach to healthcare and to integrated control of outbreaks, enhanced port health services and better personal hygiene. There is the hope that the higher index of suspicion among health workers, communities and non-governmental organizations will be maintained. Ebola is over for now in Nigeria, and there is continued decline in cases with Liberia yet to record a new case in more than 7days in March 2015 [10]. However, after EVD, what next? There is the hope that we learn from this outbreak to prevent subsequent outbreaks.
